# Leaving no one behind: Is the achievement of the Sustainable Development Goals possible without securing the dignity, rights, and well‐being of those who are “invisible”?

**DOI:** 10.1002/ijgo.13031

**Published:** 2020-01-13

**Authors:** Erin Anastasi, Bridget Asiamah, Geeta Lal

**Affiliations:** ^1^ Technical Division Campaign to End Fistula, Maternal and Newborn Health Thematic Fund United Nations Population Fund New York NY USA

**Keywords:** Obstetric fistula, Sustainable Development Goals, United Nations resolution on obstetric fistula

## Abstract

The persistence of obstetric fistula—a devastating childbirth injury occurring largely among poor, marginalized women and girls—constitutes a human rights violation and a public health crisis. The Sustainable Development Goals (SDGs) aim to “leave no one behind.” Failing to eliminate fistula jeopardizes attainment of several of the SDGs. Member States of the United Nations adopted a UN Resolution on ending fistula in 2018, calling for an end to fistula within a decade. Building upon recommendations of the UN Secretary General's 2018 Report on Obstetric Fistula, the Resolution calls for significantly increased commitments and investments to end fistula. Crucial interventions for eliminating fistula include high‐quality, equitable, accessible health systems; implementing costed national strategies for eliminating fistula; integrating fistula into national plans to achieve the SDGs; strengthening national fistula task forces; and significantly increased, sustained financial support. Fistula elimination necessitates protecting women's/girls’ human rights and addressing social determinants that affect women's/girls’ ability to “survive, thrive and transform,” including social and economic inequities; gender‐based violence; child marriage and early childbearing; and access to education. Enhanced awareness‐raising and advocacy; improved research, data, monitoring and evaluation; holistic social reintegration and survivor empowerment; and community engagement are additional key strategies for realizing this ambitious goal.

## Abstract 2

From a rights perspective, ending fistula is critical to achieving the SDGs, as called for by the UN Member States and Campaign to End Fistula.

## 1 A QUESTION OF RIGHTS

According to United Nations (UN) Resolution A/HRC/RES/39/10: “Preventable maternal mortality and morbidity and human rights in humanitarian settings,” adopted at the 39th session of the Human Rights Council in September, 2018 “…Violations of the right of everyone to the enjoyment of the highest attainable standard of physical and mental health… can cause high levels of maternal morbidity, including obstetric fistula, leading to ill health and death for women and girls of childbearing age in many regions of the world, and particularly in humanitarian settings…” Therefore, “…a dramatic and sustainable scaling up of quality treatment and healthcare services, including high‐quality emergency obstetric services and also of the number of trained, competent fistula surgeons and midwives, is needed to significantly reduce maternal and newborn mortality and to eradicate obstetric fistula.”^1^

This year's (2019) theme for the International Day to End Obstetric Fistula, “Fistula is a human rights violation—end it now!” is a call to action to end this global social injustice and health and human rights tragedy. The very existence of fistula is a serious violation of human rights and an affront to the right of all persons to hope, healing, and dignity. Fistula survivors are living reminders of health systems’ failure and a tragic sign of inequity. All too often, however, they are “invisible”—hidden away and forgotten.

## 2 LEAVING NO ONE BEHIND

The 2030 Agenda for Sustainable Development/Sustainable Development Goals (SDGs) represents a unique opportunity to promote human rights, equality, and well‐being for all.^2^, ^3^ This bold, visionary global agenda aims to “leave no one behind” and “reach the furthest behind first,” with a view toward eliminating all forms of extreme human suffering. Women and girls with obstetric fistula are surely among the furthest left behind. They have needlessly suffered some of the worst human misery imaginable. Eliminating fistula is a key element of “leaving no one behind” and the world risks failing to achieve the SDGs, especially Goals 1 (poverty); 3 (health); 4 (education); 5 (gender equality); and 10 (inequalities), if there are still women and girls left in the world suffering from fistula who are being neglected. Therefore, the UN has called on the global community to eliminate this tragic, yet preventable and largely treatable condition.

As evidence suggests, policies and programs often fail to reach the poorest and most vulnerable women and girls.^4^ These marginalized groups tend to remain invisible to policymakers, healthcare providers, and society in general. By failing to understand and address the multidimensional barriers and deeply ingrained structural violence faced by these poorest, most at‐risk groups, we risk perpetuating “invisibility, inferiority, and powerlessness,” and failing to achieve “the world we want,” as enshrined in the vision of the SDGs.

## 3 THE UN CALL TO END FISTULA WITHIN A DECADE

Although significant progress has been made in efforts to end fistula, including increased political awareness; initiatives at the global, regional, and country levels; strengthened partnerships and coordination mechanisms for fistula and maternal and neonatal health; and increasing country ownership and leadership, significant challenges remain. A new UN Resolution on fistula was adopted by Member States in December 2018. Building upon the recommendations of the UN Secretary General's 2018 Report on Fistula, the Resolution has called for significantly increased commitments and investments to end fistula “within a decade”.^5^ This represents a major and urgent shift from the 2016 UN vision of ending fistula within a generation. Both the report and the resolution link the call to end fistula with the achievement of the SDGs; integrating fistula with global efforts to achieve universal access to emergency/essential surgery; addressing root causes of fistula; and calling for UNFPA/Campaign to End Fistula, together with member states and key strategic partners, to lead the development of a new global road map to end fistula within a generation.

The UN Resolution further calls on every fistula‐affected nation to develop an inclusive costed, time‐bound national strategy and action plan to end fistula by 2030, and establish a national ministry of health/government‐led task force on fistula to bring together all key stakeholders to develop and implement this critical strategy and action plan. This is an essential component of an overall strategy to ensure universal health coverage (including access to quality sexual and reproductive health and rights) and achieve the SDGs. Key to this achievement is for governments to increase national budgets and harness domestic resources to address fistula through a multisectoral approach. In addition, the global community needs to urgently and dramatically intensify financial and technical support to nations with the greatest need.

## 4 WHAT MUST BE DONE?

To end fistula, we must ensure universal access to quality sexual and reproductive health services and comprehensive health care including fistula treatment. Crucial interventions that will contribute to eliminating fistula and to making the “invisible” women and girls affected by fistula, visible, include:

- Strengthening health systems
- Implementing and monitoring costed national strategies for eliminating fistula
- Integrating fistula into national SDGs implementation plans and operational processes to achieve the SDGs
- Strengthening national task forces for fistula
- Securing significantly increased, predictable, sustained, and adequate financial support (ensuring that a higher proportion of resources reach young women and girls)
- Strengthening awareness‐raising and advocacy
- Improving research and data
- Holistic social reintegration and follow‐up and empowerment for survivors (including engaging them as advocates, enhancing their voices and leadership)
- Increased focus on social determinants that affect the well‐being of women and girls, including eliminating gender‐based social and economic inequities; preventing child marriage and early childbearing; promoting education and broader human rights
- Fostering community participation in finding solutions, including through the active involvement of men as well as seeking the help of fistula survivors as advocates
- Monitoring and evaluation of programs to end fistula.

To end fistula within a decade, every new case of fistula should be prevented and every woman or girl suffering from fistula should receive the treatment, follow‐up, social reintegration, and rehabilitation support she needs. Prevention is the “best medicine” and is key to ending fistula. The same interventions that prevent fistula could also save many of the women and babies who die or are stillborn each year due to complications of pregnancy or childbirth. Women with fistula most often suffer the “double tragedy” of losing their babies, who are stillborn or die soon after birth.^6^

Healthy women mean healthier infants, children, families, communities, and societies. Yet, millions of women and girls still cannot access sexual and reproductive health information and services and exercise their rights. The UNFPA is the leading UN agency for sexual, reproductive, maternal, newborn, and adolescent health, galvanizing global commitment to end unmet need for family planning, end preventable maternal deaths, and end gender‐based violence and harmful practices against women and girls. UNFPA has launched a strategy to promote and protect maternal/newborn survival and well‐being, including ending fistula. This strategy includes: family planning to prevent unintended pregnancies and enable women to space their pregnancies in a healthy manner; quality, skilled, accessible, and culturally appropriate care for all women during pregnancy, delivery, and the postnatal period (including through its global midwifery program, which is active in over 120 countries); and equitable access to high‐quality, timely, emergency obstetric and newborn care for those who need it.

UNFPA has helped transform thousands of lives by supporting more than 105 000 surgeries to repair fistula. And Campaign to End Fistula partners have supported thousands more. Yet, despite remarkable progress since the Campaign's launch in 2003, we remain far from the goal of preventing every single case of fistula from occurring in the first place and from reaching, treating, and supporting the countless hundreds of thousands of women and girls around the world who are already suffering from fistula and who continue to wait in agony and suffer in silence. Tragically, at the current rate of progress, many women and girls may die without ever being treated.

## 5 TO SUCCEED OR NOT TO SUCCEED: THE CHOICE IS OURS

As highlighted in the *Lancet* article “High‐quality health systems in the Sustainable Development Goals era: time for a revolution,” high‐quality health care for all is achievable and a sensible investment, but the political will to make it happen is essential.^7^ If health systems delivered high‐quality care to all those in need, it is estimated that over 8 million lives could be saved each year in low‐ and middle‐income countries. Yet, sadly, people continue to receive poor‐quality care, and the situation is worst among the poorest and most vulnerable.

While obstetric fistula typically affects the poorest and most marginalized women and girls around the world, these women and girls are not powerless. They are strong, resourceful, and resilient. They just need to be given a chance… a chance to have their human right to health, dignity, and bodily integrity respected. A chance for an education and a decent livelihood. A chance to benefit from high‐quality, life‐saving health care, and social protection when they need it. As in the case of Ms Fiona Kevin Nalubwama, a fistula survivor from Uganda who (with support from the UNFPA/Campaign to End Fistula and Operation Fistula), after years of struggling with poverty, indignity, and near despair, recently enrolled in a school to become a midwife. This is what the SDGs are all about.

The world is at a critical crossroad in the struggle to achieve equality, dignity, and social justice for all. Those who yield power (politically, financially) hold within their hands the ability to make a difference and to heal and rectify centuries of discrimination against the poor and vulnerable. The time is now.^8^

## AUTHOR CONTRIBUTIONS

EA conceived the article and prepared the first draft. BA contributed to writing key sections of the manuscript and GL added key inputs and revised the draft. All authors revised and approved the final manuscript.

## CONFLICTS OF INTEREST

The authors have no conflicts of interest.
